# Correction of Fanconi Anemia Group C Hematopoietic Stem Cells Following Intrafemoral Gene Transfer

**DOI:** 10.1155/2010/947816

**Published:** 2010-03-16

**Authors:** Ouassila Habi, Johanne Girard, Valérie Bourdages, Marie-Chantal Delisle, Madeleine Carreau

**Affiliations:** Unité de Recherche en Pédiatrie, Department of Pediatrics, Université Laval, CHUQ-CHUL, RC-9800, 2705 Boulevard Laurier, Québec, QC, Canada G1V 4G2

## Abstract

The main cause of morbidity and mortality in Fanconi anemia patients is the development of bone marrow (BM) failure; thus correction of hematopoietic stem cells (HSCs) through gene transfer approaches would benefit FA patients. However, gene therapy trials for FA patients using ex vivo transduction protocols have failed to provide long-term correction. In addition, ex vivo cultures have been found to be hazardous for FA cells. To circumvent negative effects of ex vivo culture in FA stem cells, we tested the corrective ability of direct injection of recombinant lentiviral particles encoding FancC-EGFP into femurs of *FancC* ^−/−^ mice. Using this approach, we show that *FancC* ^−/−^ HSCs were efficiently corrected. Intrafemoral gene transfer of the *FancC* gene prevented the mitomycin C-induced BM failure. Moreover, we show that intrafemoral gene delivery into aplastic marrow restored the bone marrow cellularity and corrected the remaining HSCs. These results provide evidence that targeting FA-deficient HSCs directly in their environment enables efficient and long-term correction of BM defects in FA.

## 1. Introduction

Fanconi anemia (FA) is a rare autosomal or X-linked genetic disease manifested by early bone marrow (BM) failure, congenital abnormalities and an increased risk of cancer [1–3]. Currently, FA is defined by 13 genes/complementation groups (FA from A to N) [4], and although the specific function of each FA protein is largely unknown, they cooperate in a common cellular pathway by forming protein complexes involved in DNA damage responses, apoptosis, and stem cell function [3, 5, 6]. The primary clinical phenotype and major cause of death in patients is the progressive depletion of hematopoietic stem cells (HSCs) leading to BM failure. While the long-term curative treatment of the hematological manifestation of the disease is allogeneic BM or cord blood stem cell transplantation, this procedure carries a substantial risk with HLA-matched unrelated BM donors [7–9]. Furthermore, FA patients are hypersensitive to the conditioning regiment and present a higher incidence of secondary malignancies. Thus an alternative curative treatment for FA patients might be gene transfer into HSC. Since stem and progenitor cells can easily be harvested from the BM or peripheral blood, ex vivo transduction protocols using viral vectors as a delivery system have been attempted in FA patients but have failed to provide long-term hematopoietic reconstitution from gene-corrected cells [10, 11]. Several reasons may account for the poor success rate of these trails, for instance, the low availability of HSC found in FA patients even before severe pancytopenia [10], the reduced reconstitution ability and self-renewal capacity of FA-deficient HSC following culture [12, 13], the development of aberrant clones of FA-deficient HSC following ex vivo culture [13], as well as defective homing properties of FA-deficient BM cells [14]. Although rapid transduction of FA cells using short-term culture [15] or culture in the presence of an antioxidant [16] improved FA cell survival, these approaches still make use of cytokines and growth factors that may affect long-term HSC function [12, 17]. Thus, targeting HSC directly in their environment would ensure maintenance of their function and enable the correction of the remaining stem cells. The feasibility of in vivo gene transfer by direct intrafemoral, or intrabone marrow, injection of adeno-, retro-, and lenti-viral particles into mice has recently been demonstrated [18, 19]. These studies showed efficient transduction of HSCs with detection of the GFP transgene in progenitors and cell lineages. Using this in vivo gene delivery approach, we present data showing efficient transduction of HSC and long-lasting *FancC* transgene expression in *FancC* ^−/−^ mice following intrafemoral (IF) injections of recombinant lentiviral vector (LV). In addition, we show that transduced HSC retain their potential to differentiate into all blood cell lineages and maintain their reconstitution ability as shown by primary transplants. In vivo delivery of *FancC-*coding LV particles fully restored resistance to DNA crosslinking agents and prevented BM failure in *FancC* ^−/−^ mice. Furthermore, reversal of aplasia was observed following IF injection of the *FancC* transgene into BM-depleted *FancC* ^−/−^ mice. This preclinical study supports in vivo gene delivery of recombinant LV particles as a means to treat the hematopoietic defect in FA patients.

## 2. Methods

### 2.1. Lentivirus Production

The three-plasmid expression system containing the packaging plasmid pCMVR8.91 providing the Gag, Pol, Tat, and Rev proteins, the envelope-coding plasmid pMD.G providing the vesicular stomatitis virus glycoprotein (VSV-G), and the transfer vector pSinPGK-EGFP was a gift from D. Trono [20]. The murine FancC gene was cloned in fusion to the Enhanced Green fluorescent protein (EGFP; pEGFP-C1; Clontech) and subcloned into the LV transfer plasmid (pSIN-FancC-EGFP). Functionality of the FancC-EGFP fusion protein was tested in FANCC mutant lymphoblastoid cells (HSC536; gift from Dr. M. Buchwald) and found to fully correct the DNA crosslink sensitivity (Figure 1(a)). LV particles were produced by triple-transient Ca_3_PO_4_ transfection into HEK293T cells. Supernatants containing particles were collected, filtered, and concentrated by ultracentrifugation (90 minutes at 30,000 rpm). The infectivity of concentrated viral vector stocks was determined on HeLa cells and scored by FACS analysis for EGFP expression. Titers obtained ranged between 7 to 10 × 10^7^ transduction units per mL.

### 2.2. Intrafemoral Injections and Flow Cytometric Analysis

IF injection of LV particles was performed according to the IF cell transplantation procedure described by Mazurier et al. [21]. Briefly, 3- to 5-month-old wild type, *FancC* ^−/−^, or *FancA* ^−/−^ mice (C57BL/6J, 11th generation of backcrosses; CD45.2^+^) were anesthetized and 25 uL of concentrated LV supernatant (1.8 to 2.5 × 10^6^ transduction units per femur) were injected through the joint into the right femur using a 28.5-gauge needle. In some experiments, mice were preconditioned with MMC (0.3 mg/kg) five days prior to IF injection to induce BM aplasia as described previously [22]. To determine the correction potential of the FancC-EGFP transgene, injected mice were subjected to weekly s.c. injection of MMC (0.3 mg/kg) as previously described [22]. To evaluate the transduction efficacy, peripheral blood and BM cells were collected at various time points and analyzed by multiparameter flow cytometry (FACS Calibur cytometer; BD Biosciences) for FancC-EGFP expression and lineage markers (CD11b-PerCP or CD45R-PerCP and Ly-6G-APC or CD5-APC; BD Biosciences). All animal procedures were performed according to protocols approved by the Animal Care Committee of Laval University, Québec, Canada.

### 2.3. Transplantation Procedures

To determine the reconstitution potential of transduced HSC, IF-injected mice were sacrificed, and BM cells were collected at 4 months post injection or following 15 weeks of MMC treatment and were transplanted (2 × 10^6^ cells) into lethally irradiated recipient mice (B6.SJL-PtrcaPep3b/BoyJ; CD45.1). Reconstitution ability of donor cells was monitored as previously described [12]. Briefly, peripheral blood cells were collected once per month for 4 months. White blood cells were stained with CD45.2-PE (donor origin), CD11b-PerCP (monocytes), or CD45R-PerCP (B lymphocytes) and Ly-6G-APC (granulocytes) or CD5-APC (T lymphocytes) and analyzed by multiparameter flow cytometry. Recipient mice received antibiotics one week prior to irradiation and transplantation. 

### 2.4. Colony Forming Cell Assay

Hematopoietic committed progenitor cell assays were performed as described previously [22]. Briefly, BM cells were collected from femurs of injected or transplanted mice where 2 to 5 × 10^4^ cells per mL were seeded in complete methylcellulose medium according to the manufacturer (Stem Cell Technology) and incubated for seven to 10 days at 37°C, 5% CO_2_. Total colonies were counted and depicted as colony forming cells (CFCs). For correction potential of transduced cells, 5 nM MMC was added to the methylcellulose cultures at plating as previously described [22]. 

### 2.5. Histological Analysis

Mice were euthanized and tissues were immediately collected and placed in 10% neutral buffered formalin. Fixed tissues were embedded in paraffin, sectioned at 4 *μ*m, and stained with hematoxylin-eosin using standard methods. For the detection of transduced cells, immunohistochemistry was performed on tissue sections from gonads, liver, spleen, and thymus. Breifly, sections were deparaffinised, rehydrated, and incubated overnight with an anti-GFP rabbit polyclonal antibody (Invitrogen) diluted 1 : 3000. Antibody detection was carried out with the IDetect Super Stain System (IDLabs) and counterstained with Mayer's hematoxylin. Tissue sections were visualized at magnification of 200× using a Nikon E800 microscope equipped with a CCD camera (Hamamastu Orca ER; Nikon).

## 3. Results

### 3.1. Stable Expression of the FancC-EGFP Transgene following Intrafemoral Injection of Recombinant Lentiviral Particles

To determine the feasibility of in vivo BM stem cell gene transfer as a means to correct the Fanconi anemia stem cell defect, we first designed an HIV-derived LV coding for the murine FancC transgene in fusion with the EGFP reporter protein. This FancC-EGFP fusion construct was tested and found to phenotypically correct FANCC mutant cells (Figure 1(a)). We next explored the potential of in vivo BM cell gene transfer through IF injections of recombinant LV particles coding for EGFP directly into femurs of adult wild type and *FancC* ^−/−^ mice. EGFP expression was analyzed in peripheral blood at two weeks after injection. Cytometric analysis of peripheral blood showed 5% to 10% of EGFP-positive cells in only a few animals (4 out of 8 mice; data not shown). Cytometric analysis of peripheral blood was then performed each month. Levels of EGFP-positive cells in each animal did not change before the third month. All mice were then sacrificed at 5 months following IF injections, and surprisingly, all mice showed EGFP-positive cells in their blood and BM ranging from 5% to 50% of cells validating this technique (Figure 1(b)). No side effects were noted in IF-injected animals at any time during the procedure. 

To determine the corrective ability of intrafemoral gene transfer, recombinant *FancC*-EGFP LV particles were injected directly into femurs of adult wild type, *FancC* ^−/−^, and *FancA* ^−/−^ mice.  Figure 2(a) shows the experimental design where 6 to 10 mice from each genotype were injected with recombinant *FancC*-EGFP LV particles. Peripheral blood cells from IF-injected mice were analyzed each month for EGFP expression. Low levels of transgene expression were found in blood cells from all mice at one month following injection ranging from 5% to 20% (Figure 2(b)). The mean number of EGFP-FancC expressing cells became higher over time in all mice but more dramatically in *FancC* ^−/−^ mice (mean of 42% at 4 months) compared to WT or *FancA* ^−/−^ mice (mean of 10% and 12%, resp.). In addition, *FancC*-EGFP transgene expression was detected in both myeloid and lymphoid blood cell lineages from all injected mice suggesting that progenitors were efficiently transduced (Figure 2(c)). 

### 3.2. Intrafemoral Gene Transfer Prevents Mitomycin C-Induced Bone Marrow Failure in *FancC* ^−/−^ Mice

To determine the corrective potential of intrafemoral gene transfer into BM cells, half of the IF-injected mice from each genotype (see experimental design in Figure 2(a)) were treated weekly with 0.3 mg/kg mitomycin C (MMC), a dose known to induce progressive BM failure in FA mutant mice [22]. Wild type mice were used as positive controls and *FancA* ^−/−^ mice as negative controls since they were injected with an uncomplementing gene. MMC treatment was started seven weeks after IF-mediated FancC-EGFP gene transfer. FancC-EGFP expression in peripheral blood cells was measured before MMC treatment and every month following treatment. All mice showed low levels of FancC-EGFP-positive cells before the start of MMC treatments (Figure 3(a)). FancC-EGFP-positive cells slightly increased in WT mice after the first MMC treatment (two months post-IF injections), but dramatically increased in all *FancC* ^−/−^ IF-injected mice compared to both WT and *FancA* ^−/−^ mice. 

We have previously shown that *FancC* ^−/−^ mice of mixed background died within eight weeks of MMC treatment [22], whereas *FancC* ^−/−^ mice of C56BL/6 background died within 10 weeks (Control FancC; Figure 3(b)). We found that all IF-injected *FancC* ^−/−^ mice survived the 15 weeks of MMC treatment thus, confirming the protective effect of IF-mediated gene transfer into BM cells (Figure 3(b) and Table 1). As expected, *FancA* ^−/−^ mice injected with *FancC*-EGFP recombinant LV particles died of MMC-induced BM failure following eight weeks of MMC treatment for lack of correction. BM sections from MMC-treated mice showed that IF injection of *FancC*-EGFP prevented BM aplasia in *FancC* ^−/−^ mice and maintained the presence of all cell types including megakaryocytes (Figure 3(c)). As expected, a marked reduction in cellularity with depletion of all cell types was observed in *FancA* ^−/−^ mice whereas no remarkable effects were observed in wild type mice. This dramatic reduction in BM cellularity resembled the long-term chronic exposure to low doses of MMC in FA mutant mice as previously reported [22]. 

### 3.3. Functional Correction of Progenitors and Stem Cells from *FancC* ^−/−^ Mice

To determine if phenotypic correction occurred at the level of progenitors and stem cells, CFC assays were performed using total BM cells collected from IF-injected mice at 4 months following IF-injection or after chronic (15 weeks) MMC treatments. Results show that *FancC* ^−/−^ progenitors obtained from IF-injected mice were corrected for their MMC sensitivity showing wild type levels of CFCs in the presence of MMC suggesting that progenitors were corrected for their MMC sensitivity (Figure 4(a)). Chronic exposure to MMC has been previously shown to dramatically reduce *FancC* ^−/−^ BM CFC levels to less than 1% of normal [22]. CFC assays performed using total BM cells derived from MMC-treated FancC-EGFP-injected *FancC* ^−/−^ mice showed CFC levels similar to those of wild type mice indicating that progenitors were resistant to chronic exposure to MMC (Figure 4(b)).

To determine if the phenotypic correction also occurred at the level of repopulating stem cells, transplantation procedures were performed using purified unfractionated BM cells obtained from wild type, *FancC* ^−/−^, and *FancA* ^−/−^ IF-injected mice and from mice that survived chronic MMC treatment. FancC-EGFP protein expression was measured in total BM cells before transplants and found in the BM of all IF-injected mice (Figures 4(c) and 4(d)). FancC-EGFP protein expression was detected in Sca1-positive BM cells suggesting that progenitor/stem cells were transduced (Figures 4(e) and 4(f)). Two million of total BM cells were transplanted into lethally irradiated recipient congenic mice. Chimerism expressed as CD45.2-positive cells was detected in all recipients, those that received BM cells from untreated (Figure 5(a)) and from MMC-treated mice (Figure 5(b)). Expression of *FancC*-EGFP was detected in the majority of peripheral blood cells of donor origin (Figure 5(c)). *FancC*-EGFP transgene expression in peripheral blood cells was maintained throughout the observation period in all recipients indicating that intrafemoral gene transfer efficiently targeted the repopulating stem cells. In addition, these results suggest that the targeted stem cells were able to sustain chronic MMC treatment. 

### 3.4. Correction of Hematopoiesis in *FancC* ^−/−^ Mice with Bone Marrow Aplasia

Since FA patients were found to have reduced numbers of stem/progenitor cells early in the disease possibly before pancytopenia [10], we tested the corrective potential of IF-mediated gene transfer into a more clinically relevant disease setting. Thus, *FancC* ^−/−^ mice were preconditioned with one MMC injection (0.3 mg/kg) one week prior to IF-mediated FancC-EGFP gene transfer (see experimental design in Figure 6(a)). We previously showed that one MMC treatment in *FancC* ^−/−^ mice reduced the BM cellularity by 70% [22], decreased the short-term reconstituting cells (Lin^−^c-Kit^+^Sca1^+^CD34^+^ stem cell population) by 85% as compared to untreated mice, and decreased colony forming cells (CFCs) and long-term culture-initiating cells (LTC-IC) to levels of 16% and 33% of untreated mice, respectively [23]. Wild type, *FancC* ^−/−^, and *FancA* ^−/−^ pre-conditioned mice (8 to 12 mice per group) were IF-injected with recombinant FancC-EGFP LV particles. FancC-EGFP expression was monitored monthly over a period of four months in the peripheral blood of injected animals. FancC-EGFP transgene expression was detected in all IF injected mice (Figure 6(b)). FancC-EGFP positive peripheral blood cells increased over time to more than 90% in *FancC* ^−/−^ mice at four months following IF injections. This increase in FancC-EGFP positive cells was also observed in *FancA* ^−/−^ mice possibly due to transduction of the remaining BM stem/progenitor cells and/or clonal amplification. Concomitant with high numbers of FancC-EGFP positive peripheral blood cells, cells from both myeloid and lymphoid lineages expressed the FancC-EGFP transgene (Figure 6(c)). 

To determine the corrective potential of IF-mediated gene transfer into aplastic marrow, half of the pre-conditioned wild type, *FancC* ^−/−^, and *FancA* ^−/−^ mice were submitted to chronic MMC exposure at seven weeks following IF injection of FancC-EGFP recombinant LV particles. FancC-EGFP expression was monitored monthly over a period of 15 weeks in the peripheral blood (Figure 7(a)), with all injected animals presenting FancC-EGFP-positive blood cells over the four months period. Variation in levels of cells expressing the transgene was observed in mice from all genotypes used. All wild type and *FancC* ^−/−^ mice survived the chronic exposure to MMC while *FancA* ^−/−^ mice died between eight and 11 weeks following MMC treatment (Figure 7(c) and Table 1). FancC-EGFP expression was detected in all blood cell lineages tested from injected *FancC* ^−/−^ and wild type mice at 15 weeks of MMC exposure (Figure 7(b)) implying that stem/progenitor cells were efficiently transduced and corrected for their sensitivity to MMC. BM sections from preconditioned mice at four months following IF injections showed that *FancC* ^−/−^ mice, but not *FancA* ^−/−^ replenished their BM and maintained the presence of all cell types including megakaryocytes (Figure 7(d)). In addition, BM sections of animals following chronic exposure to MMC showed that IF-injected *FancC* ^−/−^, but not *FancA* ^−/−^, mice replenished their BM cellularity as in wild type mice. Furthermore, transplants performed with BM cells from these pre-conditioned mice at four months following IF injection showed that the remaining stem cells of *FancC* ^−/−^ mice with BM aplasia were transduced thus enabling them to maintain their reconstitution potential (Figure 8). Collectively, these results clearly indicate that the remaining stem/progenitor cells of an aplastic marrow can be efficiently transduced, and thus genetically corrected through IF injection of recombinant LV particles. 

## 4. Discussion

Mouse models of FA have been extensively studied in the context of gene therapy protocols using various targeting viral vectors [24–27]. All of these studies have shown that ex vivo gene transfer is feasible as treatment for the hematopoietic defects observed in FA. However, in the context of clinical gene therapy, sufficient numbers of hematopoietic stem/progenitors cells from FA patients are difficult to obtain even following G-CSF-mobilization [28]. Furthermore, ex vivo culture for gene transfer is deleterious for FA cells and in retrospect would possibly explain the poor success rate of earlier gene therapy trials in FA patients [11–13, 17]. Our data now provides an alternative to ex vivo gene transfer approaches showing the efficacy of direct intrafemoral gene delivery in correcting the hematopoietic defects in FA. 

Our results demonstrate that sustained long-term expression of the *FancC* transgene is achievable using a direct in vivo gene transfer approach. By using LV to deliver the *FancC*-EGFP transgene, hematopoietic stem and progenitor cells were efficiently transduced in their environment as shown by the maintenance of *FancC*-EGFP expression in various peripheral blood cell types from IF-injected animals over a four-month period and following transplants. 

Transduced stem and progenitor cells were functionally corrected for their MMC sensitivity as shown by wild type levels of CFC in the presence of MMC, thus showing that the inserted transgene is functional in correcting this defect. In addition, mice intrafemorally injected with the *FancC-EGFP* transgene were resistant to the MMC-induced progressive BM failure. Colony formation assays and histological analysis of BM tissues from the sternum not only confirmed the resistance to MMC treatment but also showed that *FancC* ^−/−^ mice replenished their BM. In addition, the fact that the sternum showed replenished BM cellularity indicates that corrected stem/progenitor cells migrated from the injected site to other BM sites. Analysis of transgene expression in both femurs and in the thymus and spleen (data not shown) also confirmed the migration of transduced hematopoietic cells to other hematopoietic tissues as previously reported [18, 19]. These previous gene markings studies also showed that the hematopoietic system remained the main target of IF-mediated gene transfer as compared to other nonhematopoietic tissues such as gonads where undetectable to near background levels of transgene have been reported [18, 19]. We have analyzed tissues from the liver and gonads by immunohistochemistry using an anti-GFP antibody and did not find any positive cells in the tissue sections tested (data not shown).

We used weekly injection of MMC as a measure of functional correction of transduced stem cells. This treatment is probably more deleterious in view of FA cells inability to repair MMC-induced DNA lesions than the pressure from the environment that FA patients are subjected to. Thus, resistance to chronic MMC treatment in IF-injected *FancC* ^−/−^ mice supports the notion that corrected cells have a selective advantage over noncorrected cells in maintaining hematopoiesis. 

We also applied this in vivo gene transfer approach in a relevant human disease setting where *FancC* ^−/−^ mice were preconditioned with MMC to induced BM aplasia before IF injection of recombinant FancC-EGFP LV particles. We had previously shown that MMC treatment reduces BM cellularity by 70% and both short- and long-term reconstituting HSC by 85%, thus mimicking aplastic BM found in FA patients [22]. Using this model, we were able to show that the remaining stem cells were transduced and genetically corrected for their hematopoietic defects showing multilineage reconstitution. Suprisingly, levels of EGFP-FancC positive cells increased in both *FancC* ^−/−^ and *FancA* ^−/−^ preconditioned animals over time suggesting transduction of the remaining progenitor/stem cells and consequently clonal amplification of these transduced cells. However, IF-injected aplastic *FancC* ^−/−^ mice but not *FancA* ^−/−^ that received weekly MMC treatment survived the treatment and showed replenished BM cellularity and maintained the presence of all cell types. Therefore, our results indicate that the few remaining stem cells of an aplastic mouse could be corrected through intrafemoral injection of recombinant LV, thus maintaining efficient long-term hematopoiesis. 

During the course of these experiments, we did not observe adverse effects of the procedure nor did we detect leukemia or myelodysplasia in IF-injected and transplanted mice also shown by normal BM architecture in IF-injected mice and transplanted recipients. Although leukemia has rarely been observed in FA mice, clonal evolution reminiscent of myelodysplasia has been observed in *FancC* ^−/−^ mice at 16 months following an ex vivo transduction approach using retroviral vectors [13, 29] further supporting that in vivo gene transfer into HSC in their environment prevents deleterious effects caused by ex vivo manipulation. The use of LV, which can transduced quiescent cells [30] thus precluding stem cell activation, may also be less detrimental to FA cells. Longer follow-up and studies in larger animals may be needed to rule out possible deleterious effects of insertional mutagenesis using LV-mediated in vivo gene transfer. There are several ongoing and upcoming LV-mediated gene therapy clinical trials for the treatment of various diseases including FA [31]. One such trial has shown efficacious correction of X-linked adrenoleukodystrophy and expression of the transgene at 30 months after infusion without adverse effects [32]. Although most of the current LV-mediated gene therapy trials use ex vivo transduction protocols, the clear clinical benefits recently reported suggest that LV-mediated gene transfer may be beneficial for the treatment of FA.

In conclusion, this study demonstrates that LV-mediated in vivo gene therapy for the treatment of Fanconi anemia is feasible even following BM aplasia. The resolution of the BM defect following in vivo gene delivery in preconditioned *FancC* mice provides the basis for a new alternative treatment for FA patients with limited numbers of stem/progenitor hematopoietic cells. This preclinical study therefore provides the basis for a gene transfer strategy for targeting HSC in their environment without the need for ex vivo manipulations or the need for conditioning regimen and transplantation procedures.

## Figures and Tables

**Figure 1:**
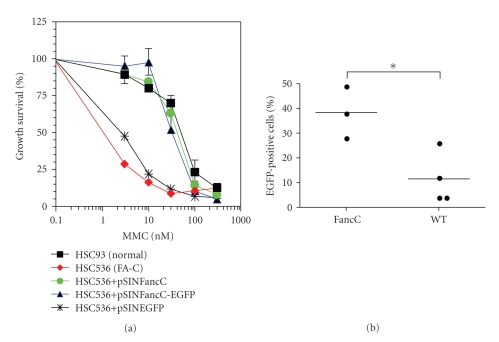
Corrective ability of the *FancC*-EGFP transgene. (a) FA group C mutant cells (HSC536) were transduced with either pSINFancC and pSINFancC-EGFP or pSINEGFP recombinant lentiviral particles. Growth survival of transduced cells as compared to normal cells (HSC93) was established with various concentrations of mitomycin C (MMC). Each point represents the mean ± SEM of three separate experiments. Absence of SEM bars represents values too low to appear in the graph. (b) EGFP transgene expression in bone marrow cells of intrafemorally injected wild type and *FancC* ^−/−^ mice five months following IF injections. Each dot represents the EGFP expression of an individual IF-injected mouse. Horizontal line: mean value. *P < .05.

**Figure 2:**
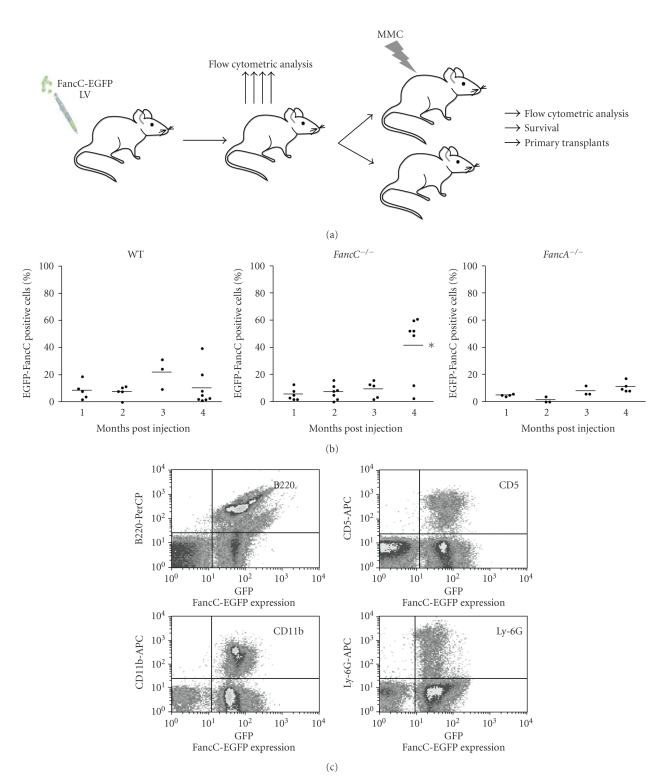
Intrafemoral gene transfer into BM of FA mutant mice. (a) Illustration of the experimental protocol used to determine the corrective potential of intrafemoral LV-mediated gene transfer into BM cells. Wild type (WT), *FancC* ^−/−^ and *FancA* ^−/−^ mice were intrafemorally injected with recombinant FancC-EGFP lentiviral particles. Transgene expression was monitored each month for at least four months. At 7 weeks following IF injections, each group of mice was divided where half of the mice were treated with MMC for 15 weeks (weakly injection of 0.3 mg/kg). (b) FancC-EGFP transgene expression in peripheral blood cells of IF injected WT, *FancC* ^−/−^, and *FancA* ^−/−^ mice as a function of time. Each dot represents the FancC-EGFP expression of an individual IF-injected mouse. Horizontal line: mean value. *P < .02. (c) Representative FACS profiles of FancC-EGFP expression in *FancC* ^−/−^ peripheral blood cell lineages at four months after IF injection.

**Figure 3:**
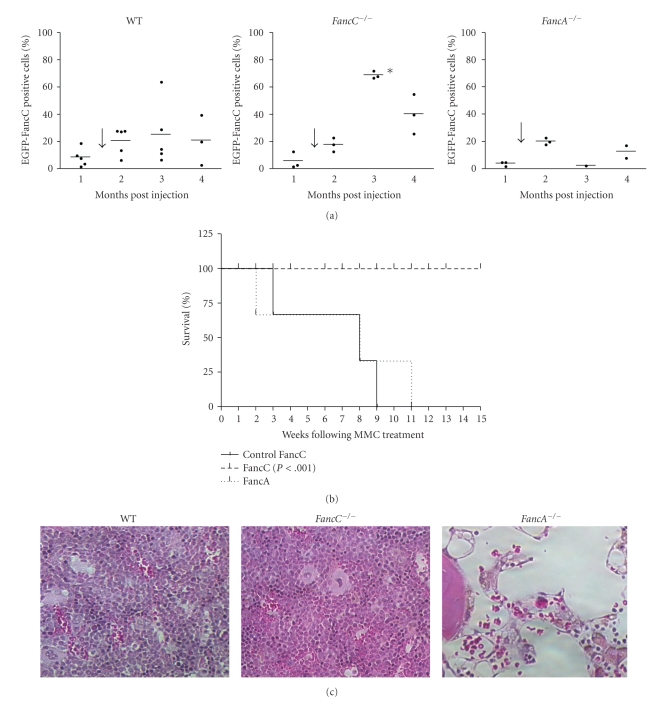
Intrafemoral gene transfer prevents MMC-induced BM aplasia. (a) FancC-EGFP transgene expression in peripheral blood cells of IF-injected wild type (WT), *FancC* ^−/−^, and *FancA* ^−/−^ mice as a function of time. Each dot represents the FancC-EGFP expression of an individual IF-injected mouse. The arrow represents the start of weekly MMC injections (0.3 mg/kg). Horizontal line: mean value. *P < .02. (b) Survival curves of IF-injected *FancC* ^−/−^ (*n* = 5) and *FancA* ^−/−^ (*n* = 3) mice following MMC treatments. Control *FancC* ^−/−^ mice represent uninjected mice treated weakly with MMC (*n* = 22). (c) Representative histological appearances of the sternum from injected mice after 15 weeks of MMC treatment. Hematoxylin-eosin staining: original magnification 200×.

**Figure 4:**
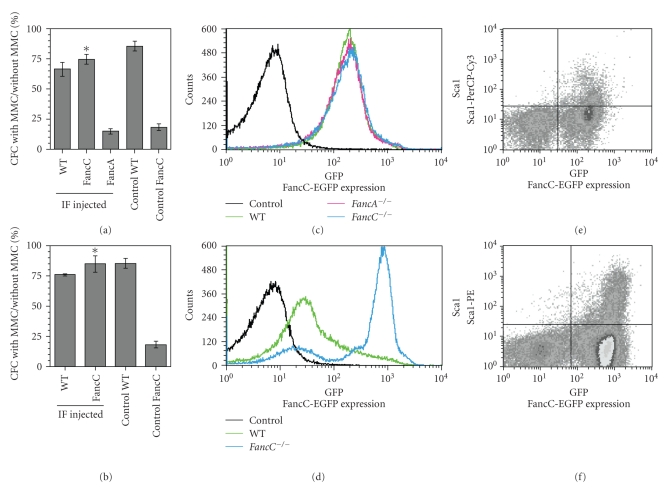
Functional correction of BM progenitors following LV-mediated *FancC-EPFP* gene transfer. CFC analysis of BM cells from IF-injected mice at four months following injection (a) and following chronic MMC treatments (b). Percent CFC numbers with MMC compared to cultures without MMC. Controls represent noninjected WT or *FancC* ^−/−^ mice. Data represent the mean ± SEM obtained from at least 3 mice in each group each done in triplicates. *P < .00005. (c) and (d) Representative cytometric profiles of FancC-EGFP expression in BM cells from IF-injected mice at four months following injection (c) and following chronic MMC treatment (d). (e) and (f) Representative cytometric profiles of FancC-EGFP expression in Sca1-positive BM cells from IF-injected *FancC* ^−/−^ mice at four months following injection (e) and following chronic MMC treatment (f).

**Figure 5:**
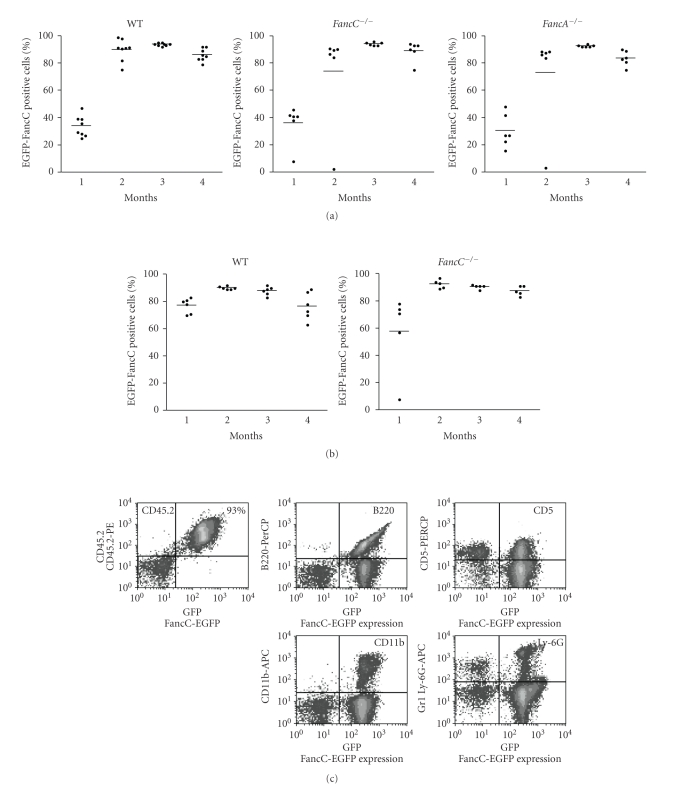
Reconstitution ability of intrafemorally transduced HSC following transplants. Percent donor chimerism (CD45.2-positive cells) in peripheral blood cells from recipients transplanted with total BM from (a) IF-injected WT, *FancC* ^−/−^, or *FancA* ^−/−^ mice and (b) from WT or *FancC* ^−/−^ mice that received chronic MMC treatments at 7 weeks following IF-injection as a function of time. Each dot represents donor chimerism of an individual recipient. Horizontal line: mean value. (c) Representative cytometric profiles of FancC-EGFP expression in peripheral blood cell lineages from transplanted mice at 3 months following transplantation with BM cells from *FancC* ^−/−^ IF-injected animals.

**Figure 6:**
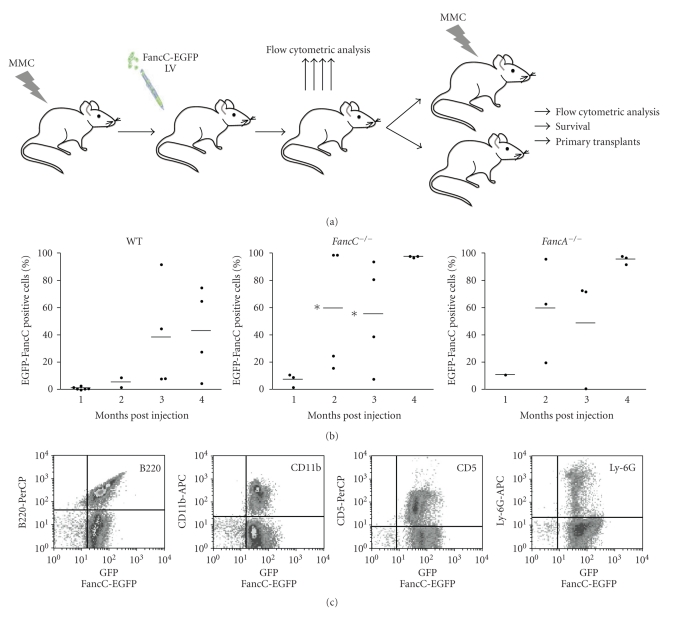
Corrective potential of intrafemoral gene transfer into FA mice with BM aplasia. (a) Illustration of the experimental protocol used to determine the corrective potential of intrafemoral LV-mediated gene transfer into aplastic BM. Wild type (WT), *FancC* ^−/−^, and *FancA* ^−/−^ mice were preconditioned with MMC five days prior to intrafemoral injection of recombinant FancC-EGFP lentiviral particles. At 7 weeks following IF injections, each group of mice was divided where half of the mice were treated with MMC for 15 weeks (weakly s.c. injection of 0.3 mg/kg). (b) Transgene expression was monitored each month for four months: FancC-EGFP transgene expression in peripheral blood cells of preconditioned IF-injected wild type (WT), *FancC* ^−/−^, and *FancA* ^−/−^ mice. Each dot represents the FancC-EGFP expression of an individual IF-injected mouse. Horizontal line: mean value. *P < .05. (c) Representative cytometric profiles of FancC-EGFP expression in peripheral blood cell lineages from *FancC* ^−/−^ preconditioned IF-injected mice at four months following in vivo gene transfer.

**Figure 7:**
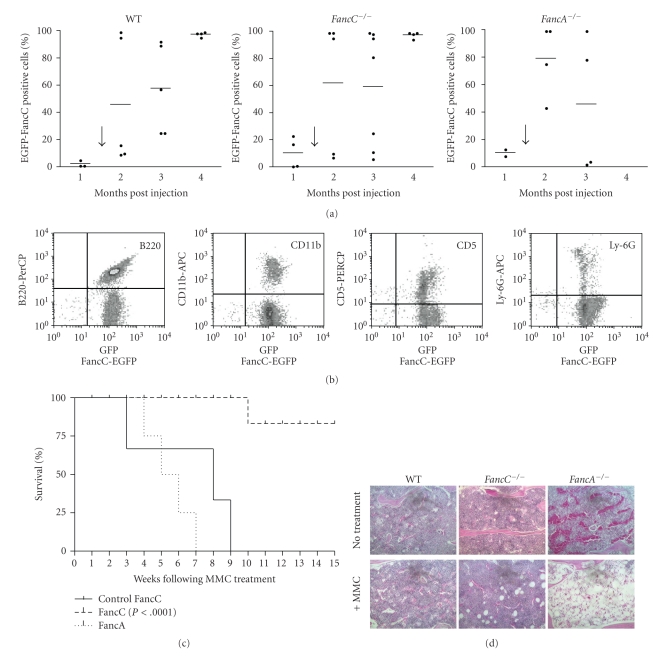
Prevention of BM failure following IF gene transfer into mice with BM aplasia. (a) FancC-EGFP transgene expression in peripheral blood cells of preconditioned IF-injected wild type (WT), *FancC* ^−/−^, and *FancA* ^−/−^ mice that received chronic MMC treatments at 7 weeks following IF-injection as a function of time. The arrow represents the start of weekly MMC injections (0.3 mg/kg). Each dot represents the FancC-EGFP expression of an individual IF-injected mouse. Horizontal line: mean value. (b) Representative cytometric profiles of FancC-EGFP expression in peripheral blood cell lineages from *FancC* ^−/−^ preconditioned IF-injected mice at 15 weeks following weekly MMC treatment. (c) Survival of preconditioned IF-injected *FancC* ^−/−^ (*n* = 6) and *FancA* ^−/−^ (*n* = 4) mice following MMC treatments. Control *FancC* ^−/−^ mice represent uninjected mice treated weakly with MMC (*n* = 22). (d) Representative histological appearances of the sternum from preconditioned and IF-injected mice at four months following IF injections (no treatment) and at 15 weeks following weekly MMC treatment (+ MMC). Hematoxylin-eosin staining: original magnification 200×.

**Figure 8:**
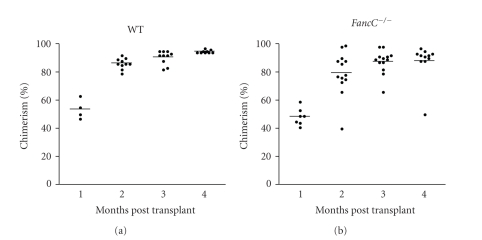
Reconstitution ability of intrafemorally transduced HSC following transplants: percent donor chimerism (CD45.2-positive cells) in peripheral blood cells from recipients transplanted with total BM from preconditioned IF-injected WT and *FancC* ^−/−^ mice as a function of time. Each dot represents donor chimerism of an individual recipient with ten to fourteen mice per genotype. Horizontal line: mean value.

**Table 1:** Number of mice that survived MMC-treatments after LV-mediated intrafemoral gene transfer.

	Wild type		*Fa* *nc* *C* ^−/−^		*Fa* *nc* *A* ^−/−^	
		Pre-conditioned*		Pre-conditioned*		Preconditioned

	5/5	6/6	5/5	6/6	3/3	3/4^*¶*^
Weekly MMC (0.3 mg /kg)^†^	5/5	6/6	5/5	5/6^‡^	0/3	0/4

Number of mice that survived/number of mice IF-injected with *EGFP-FancC* recombinant viral particles.

* Mice were pre-conditioned with MMC (0.3 mg/kg) 4 days prior to IF injection.

^†^ Weekly treatment with MMC for 15 weeks.

^‡^ 1 *FancC* ^−/−^ mouse died due to bleeding during blood collection.

^*¶*^ 1 *FancA* ^−/−^ mouse died 15 days following IF injection from a mishap unrelated to vector administration.
